# Field study on the suitability of oral fluid samples for monitoring of *Lawsonia intracellularis* and *Brachyspira hyodysenteriae* by multiplex qPCR under field conditions

**DOI:** 10.1186/s40813-024-00415-4

**Published:** 2025-01-07

**Authors:** Matthias Eddicks, Gerald Reiner, Sigena Junker, Hermann Willems, Sabrina Becker, Julia Stadler, Josefine Hagn, Mathias Ritzmann

**Affiliations:** 1 Clinic for Swine at the Centre for Clinical Veterinary Medicine, Ludwig-Maximilians-Universität München, 85764 Oberschleissheim, Germany; 2 Clinic for Swine of the Justus-Liebig-Universität Gießen, 85392 Gießen, Germany; 3 Veterinary Practice Hagn, 84076 Pfeffenhausen, Germany

**Keywords:** Screening, Enteric diseases, Swine, Fattening, Livestock

## Abstract

**Background:**

Monitoring or surveillance of infectious diseases is crucial in terms of herd health management of livestock. Investigations of oral fluids have become an animal friendly routine strategy to monitor respiratory pathogens in pigs. Less is known about the suitability of oral fluids for the detection of enteric pathogens in swine. In the present study we evaluated the use of oral fluids to monitor *B. hyodysenteriae* and *L. intracellularis* compared to pooled fecal samples by multiplex qPCR in a pen-wise follow-up of fattening pigs. Therefore, we collected oral fluids at an age of 12, 16 and 20 weeks of life and compared them to pooled fecal samples collected from the same pens on two fattening farms.

**Results:**

Cohen´s Kappa analysis revealed a substantial agreement between oral fluids and pooled fecal samples on pen level (Cohen´s Kappa: 0.745; *p* < 0.001). DNA-loads of *L. intracellularis* were tendentially higher (*p* = 0.053) in pooled fecal samples than in the corresponding OFs.

**Conclusions:**

The present study shows that oral fluids are an appropriate tool to monitor *B. hyodysenteriae* and *L. intracellularis* on conventional fattening farms under field conditions. However, multiple pen testing should be conducted to increase the diagnostic performance and sensitivity.

## Background

Oral fluids (OFs) display a user and animal friendly way to gain material for herd diagnostics, that can easily be collected due to the natural explorative behavior of swine [1]. It displays a mixture of different components including saliva, food particle, cell detritus, tracheal-nasal secretions, gastrointestinal reflux, and serum-derived compounds, reviewed by Henao-Diaz, et al. [2]. The large number of animals that can be addressed by OF sampling can, under certain circumstances, increase the diagnostic sensitivity for the detection of infectious pathogens on pen [3] or herd level [4]. OFs are most notably known for surveillance or monitoring purposes of respiratory pathogens [5–7]. Although OFs are not sufficient to gain an etiological diagnose in pigs, they are well suited to obtain a prognostic profiling in a certain herd [7]. Naturally, enteric pathogens as *Lawsonia* (*L.*) *intracellularis* and *Brachyspira* (*B.*) *hyodysenteriae* are not present within these components. Concerning the use of OFs for *L. intracellularis* diagnostics, Barrera-Zarate, et al. [8] were able to demonstrate that OFs can facilitate indirect detection of the infection, but published studies on the detection of nucleic acids of *L. intracellularis* or *Brachyspira* spp. in OFs are rare up to date. Nevertheless, oral infection seems to be the most relevant way of pathogen transmission of the aforementioned pathogens [9], indicating that they could also be detected by examination of OFs. Furthermore, OFs are often contaminated with feces or liquid manure [2], leading to the hypothesis that enteric pathogens or at least genome fragments should be present in OFs. That enteric pathogens can principally be detected in OFs was already shown by Schott, et al. [10] who evaluated OFs for the surveillance of food-borne pathogens in pig farms. The ease of gaining animal- and user-friendly sampling material for herd diagnostics increases the compliance of farmers concerning diagnostic measures. As OFs can be used for the detection of several pathogens that play a major role for the herd health, the addition of enteric pathogens as *L. intracellularis* or *B. hyodysenteriae* to an OF-screening increases the diagnostic panel for OFs and thus, delivers more relevant data within one single diagnostic approach. Thus, the objective of the present examination was to evaluate the agreement of pen-wise collected pooled fecal samples (PFS) in terms of *L. intracellularis* and *B. hyodysenteriae* DNA detection rate on two fattening farms in Germany.

## Methods

The present study was conducted on two fattening farms in Southern Germany (farm A and farm B) with a known history of *L. intracellularis* infection. Additionally, the herd attending veterinarian of farm B reported about a recent clinical outbreak of *B. hyodysenteriae* before the start of the study.

In both farms the pigs were kept in accordance with the guidelines of the German Animal Welfare Ordinance [12]. Farm A was a conventional fattening farm with a total of 1340 fattening pigs managed in a barn-wise all-in all-out workflow and a maximum of 28 pigs / pen. This farm purchased pigs 12 weeks of age from one sow herd. Vaccination with an oral life vaccine against *L. intracellularis* was carried out until six-month prior the present examination. Thus, the examined batch of pigs was not vaccinated against *L. intracellularis*. Furthermore, the pigs on this farm were vaccinated against porcine circovirus 2 (PCV2) and *Mesomycoplasma* (*M.*) *hyopneumoniae* as suckling piglets. The pigs received liquid feed via a sensor controlled short trough. The pigs were housed on concrete slatted floors; water was available ad libitum via nipple drinkers.

Farm B was a conventional fattening farm with a total of 500 fattening pigs managed in a continuous workflow with a maximum of 20 pigs / pen. This farm received pigs from different sources. However, all pigs within the study batch were from the same farm, where they received an oral live vaccine against *L. intracellularis* at three weeks of age. Further vaccinations of the piglets included vaccination against PCV2 and *M. hyopneumoniae*. Feeding was dry feed based on farm own cereals. The pigs were housed on concrete slatted floors; water was available ad libitum via nipple drinkers. Due to clinical signs of diarrhea within the 16th week of age, the herd attending veterinarian decided to treat the animal of the enrolled batch for five consecutive days with Tiamulinhydrogen-fumarate (Denagard^®^, Elanco Animal Health Deutschland GmbH, Bad Homburg, 8.8 mg/kg BDW) via the drinking water. The treatment was applied to the pigs after the collection of the OFs at 16th week of age.

### Sampling

A pre-screening for *B. hyodysenteriae*, and *L. intacellularis* was conducted prior to the start of the study (approximately four weeks in farm A and seven weeks in farm B) in animals of a preceding batch to confirm the infection status of the farms as reported by the herd attending veterinarian. Therefore, 3 freshly dropped feces were collected from 5 pens of growing-finishing pigs (approximately 17 weeks of age for farm A and 20 weeks of age for farm B) in the corresponding farms. Fecal samples from each pen were pooled (pooled fecal sample, PFS) and examined as described below. Within the subsequently conducted follow-up study one newly introduced fattening batch was included (farm A: 12 pens; farm B: 10 pens). The samples were collected at 12 weeks (directly after placement), 16 weeks and 20 weeks of age always in the same pens. From each pen one pool of freshly dropped feces of three pigs/pen was collected. If feces with a divergent consistency were present, they were sampled preferentially. Thus, in total 66 (farm A: 36; farm B 30) pooled fecal samples were available for the examinations.

OFs were collected at the same time and from the same pens as the PFS. For the collection of the OFs the Oral Fluid Sample Collection Accessory Kit (IDEXX® Laboratories Inc.) were used. The ropes were placed at pig´s shoulder height in a clean area with distance to nipple drinkers, feeding through and neighboring pigs to avoid contamination. The ropes remained in the pens for approximately 20 min. For each pen one rope was used. Subsequently, the OFs were wrung out in the provided sealable 5 ml collection container. For each cotton rope new disposable gloves were used. In total 66 OFs were collected and available for molecular biological examinations. To ensure all samples were examined within the same batch of PCR all samples (PFS and OFs) were stored frozen at −20 °C until further analysis. Time between sampling and freezing did not exceed two hours. Storage before PCR examinations was up to eight weeks. A simple feces scores (FS) was obtained for each pen at the time of sampling as following: 1: normal feces consistency in the sampled pen at time of sampling, 2: feces with soft consistency present in the sampled pen at time of sampling, 3: soupy consistency / diarrhea present in the sampled pen at time of sampling.

### Molecular biological examinations

All OFs and PFS of the study and the pre-screening were analyzed by the same batch of a multiplex qPCR as published elsewhere [11].

### Statistical analysis

Gained data was documented in Microsoft Excel as metric scale (DNA copies/g feces) and encoded in binary data (e.g., PCR positive/negative). Statistical calculations were performed with the software IBM SPSS Statistics version 28.0.1.0 for Microsoft^®^ Windows. Qualitative and quantitative PCR results were displayed descriptively. Metric data (qPCR results) were tested for normal distribution by Kolmogorov–Smirnoff Test. As the quantitative results of the qPCR were not normally distributed, the Mann–Whitney U Test was used to verify the Null Hypothesis of an equal distribution of the qPCR results over the categories Material (OFs vs. PFS). Chi2-test was conducted to evaluate associations between the detection of one pathogen in dependency of the used sample material. Odds ratio was determined to estimate the chance of detection for each pathogen and material. The interrater reliability between the materials PFS and OF to assign a pen as positive for one of the pathogens was evaluated by Cohens Kappa and interpreted as described by Landis and Koch [13]. Level of significance was* p* ≤ 0.05.

## Results

### Prescreening results

Pooled fecal samples from farm A were positive for *L. intracellularis* DNA only, whereas the examination of the pooled fecal samples from farm B revealed *L. intracellularis* and *B. hyodysenteriae* positive fecal pools (Table 1).

**Table 1:** Quantitative PCR results (DNA copies/g feces) of the pre-screening performed on farm A and B (n.d.= not detected)

Pool-number	*L. intracellularis*		*B. hyodysenteriae*	
	Farm A	Farm B	Farm A	Farm B
1	2.08 × 10^5^	7.47 × 10^4^	n.d	n.d
2	3.28 × 10^7^	n.d	n.d	5.72 × 10^5^
3	2.24 × 10^5^	2.02 × 10^4^	n.d	n.d
4	1.44 × 10^5^	n.d	n.d	n.d
5	9.60 × 10^6^	n.d	n.d	2.51 × 10^7^

### Results of the main study

**Fig. 1:**
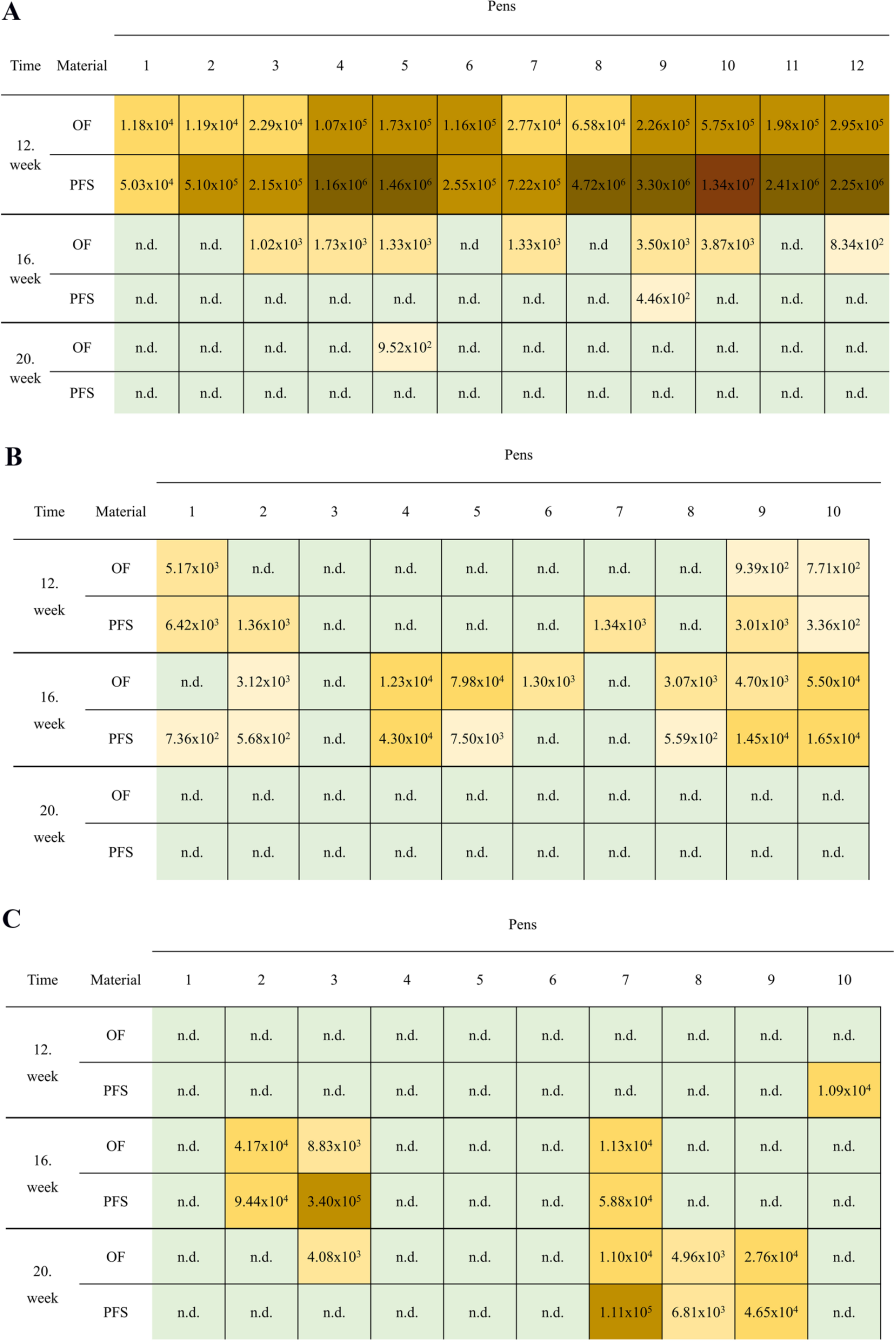
**A** Farm A: Quantitative results of the qPCR for *L. intracellularis* in PFS or OFs for each pen at different time points of sampling. Different colors indicate different DNA-loads in the sample material (green: negative, brown: the darker, the higher the DNA loads; n.d. = not detected). PCR results represent DNA copies/g feces or /ml OF. **B** Farm B: Quantitative results of the qPCR for *L. intracellularis* in PFS or OFs for each pen at different time points of sampling. Different colors indicate different DNA-loads in the sample material (green: negative, brown: the darker, the higher the DNA loads; n.d. = not detected). PCR results represent DNA copies/g feces or /ml OF. **C** Farm B: Quantitative results of the qPCR for *B. hyodysenteriae* for each pen at different time points of sampling. Different colors indicate different DNA-loads in the sample material (green: negative, brown: the darker, the higher the DNA loads; n.d. = not detected). PCR results represent DNA copies/g feces or /ml OF

In total 45.5% and 10.6% of all OFs were positive for *L. intracellularis* and *B. hyodysenteriae* DNA by qPCR, respectively. Concerning PFS, 37.9% were positive for *L. intracellularis* and 10.6% for *B. hyodysenteriae* DNA. Three pens were positive for both pathogens (Fig. 1B and C; farm B). An overview on the PCR results on pen level based on OFs and PFS is present in Table 2.

**Table 2:** Qualitative qPCR results for *L. intracellularis and B. hyodysenteriae* on pen level for both farms and all times of sampling and *p*-values for Chi2-test results

Detection rate and number of positive pens									
		*L. intracellularis* positive pens				*B. hyodysenteriae* positive pens			
Farm	Week	OFs	PFS	Total	*p*-value	OFs	PFS	Total	*p*-value
A	12	100.0% (12/12)	100.0% (12/12)	100.0% (12/12)	-	0.0% (0/12)	0.0% (0/12)	0.0% (0/12)	–
A	16	58.3% (7/12)	8.3% (1/12)	58.3% (7/12)	0.027	0.0% (0/12)	0.0% (0/12)	0.0% (0/12)	–
A	20	8.3% (1/12)	0.0% (0/12)	8.3% (1/12)	1.000	0.0% (0/12)	0.0% (0/12)	0.0% (0/12)	–
A	Total	55.6% (20/36)	36.1% (13/36)	55.6% (20/36)	0.155	0.0% (0/12)	0.0% (0/12)	0.0% (0/12)	–
B	12	30.0% (3/10)	50.0% (5/10)	50.0% (5/10)	0.650	0.0% (0/10)	10.0% (1/10)	10.0% (1/10)	1.000
B	16	70.0% (7/10)	70.0% (7/10)	80.0% (8/10)	1.000	30.0% (3/10)	30.0% (3/10)	30.0% (3/10)	1.000
B	20	0.0% (0/10)	0.0% (0/10)	0.0% (0/10)	–	40.0% (4/10)	30.0% (3/10)	40.0% (4/10)	1.000
B	Total	33.3% (10/30)	40.0% (12/30)	43.3% (13/30)	0.789	23.3% (7/30)	23.3% (7/30)	26.6% (8/30)	1.000
A + B	Total	45.5% (30/66)	37.9% (25/66)	50.0% (33/66)	0.480	10.6% (7/66)	10.6% (7/66)	12.1% (8/66)	1.000

There was no significant effect of the used materials on the odds to detect one of the two pathogens by PCR on pen level, except in week 16 on farm A when significantly more OFs were *L. intracellularis* positive than PFS (OR: 15.40; 95% CI: 1.47 – 160.97; *p* = 0.027). A detailed descriptive presentation of the qualitative and quantitative examination results over the entire study-period for each pen and sample occasion is given in Fig. 1A–C (Fig. 1A: farm A; only *L. intracellularis* positive; Fig. 1B and C: farm B; *L. intracellularis* and B*. hyodysenteriae* positive).

**Fig. 2:**
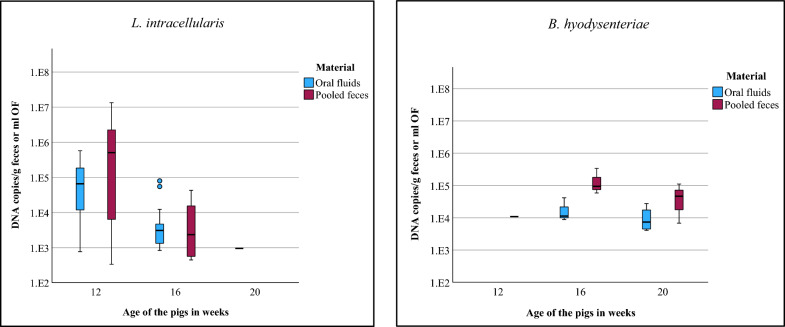
Boxplots of DNA copies/g feces or ml OF for *L. intracellularis* and *B. hyodysenteriae* in oral fluids (OFs) and pooled feces samples (PFS) over the entire study period of both study farms. Only PCR positive results included

Although DNA loads of *L. intracellularis* were numerically higher in PFS (mean: 1.22 × 10^6^) than in OFs (mean: 6.70 × 10^4^) this difference was not statistically significant (*p* = 0.053). Concerning *B. hyodysenteriae* no significant differences concerning the DNA-loads in PFS (mean: 9.55 × 10^4^) and OFs (mean: 1.56 × 10^4^) were detected. The results of the qPCR at the three different times of sampling are depicted in Fig. 2.

To evaluate the agreement of both sample types on pen level for *L. intracellularis* or *B. hyodysenteriae* we calculated the Cohen´s Kappa over the entire study period for both farms and for each single time point of sampling and farm. This calculation revealed a substantial agreement for the status “pen *L. intracellularis* positive” and a close to perfect agreement for the status for “pen *B. hyodysenteriae* positive” for the entire study period and each farm. However, the results of the Cohen´s Kappa under respect of the different time points of sampling revealed a high variability. The detailed results concerning the interrater reliability are presented in Table 3.

**Table 3:** Cohen´s Kappa for: “pen *L. intracellularis* positive” or “pen *B. hyodysenteriae* positive” under consideration of both farms, time of sampling and total (n.d.: not detected)

		*L. intracellularis*	*B. hyodysenteriae*
		Cohen´s Kappa	Cohen´s Kappa
Farm A	Total	0.623	n.d.^*^
	W12	1.000	n.d
	W16	0.122	n.d
	W20	1.000	n.d
Farm B	Total	0.714	0.814
	W12	0.600	0.895
	W16	0.524	1.000
	W20	1.000	0.738
Farm A + B	Total	0.659	0.840

### Fecal scores

**Fig. 3:**
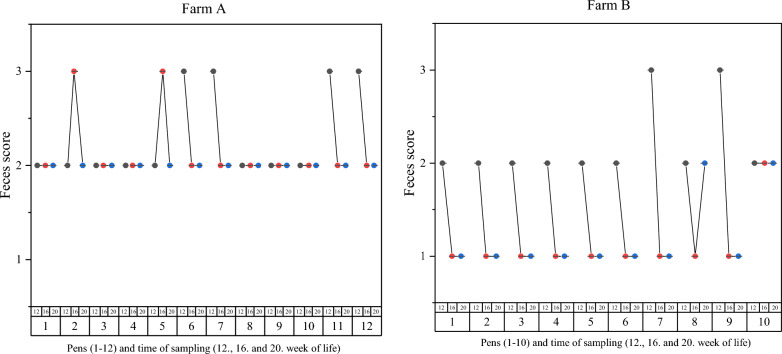
Feces scores of farm** A** and farm** B** for each time of sampling per pen over the entire study period

In farm A, the mean feces score over the entire study period (FS: 2.16) was significantly (*p* < 0.001) higher compared to farm B (FS: 1.5). Under respect of the farm and considering both pathogens (where applicable), no significant associations were present between the feces score per pen and the DNA loads in the samples. Moreover, the chance to detect *L. intracellularis* or *B. hyodysenteriae* was not significantly associated with the feces score in the corresponding pens. The feces scores per pen at all times of sampling of farm A and farm B are shown in Fig. 3.

## Discussion

The present study was conducted on two farms with a known history of *L. intracellularis* or *B. hyodysenteriae* infection. This status was confirmed by a diagnostic screening prior the start of the study. Farm A revealed a *L. intracellularis* and farm B a *L. intracellularis* and *B. hyodysenteriae* positive screening result. From that point of view, our study population seemed to be adequate concerning the objectives of the study referring to the beforementioned pathogens. However, due to the limited number of farms and sample size, our study has explorative character and large-scale studies are necessary to generalize the present findings.

Meanwhile qPCR is very well established, both as mono-PCR and as multiplex PCR for rapid analysis of *Brachyspira* spp. and *L. intracellularis* with increased sensitivity compared to normal PCR and the simultaneous possibility of quantifying the pathogens [11, 14–17]. The multiplex approach offers a good possibility to detect several pathogens synchronously during screenings and to reduce the diagnostic workload. For the investigation of intestinal pathogens or pathogens shed by feces, pooled fecal samples (PFS) provide accessible sample sources at any time. Particularly for PCR analyses, the freshness of the samples is of secondary importance, so that the animals do not have to be fixed for rectal sampling, which reduces stress in the sampled population. However, when detecting microbial nucleic acids by PCR, false negative results can always be a result of inhibitors present in the feces of the animals [18, 19]. This aspect can play an essential role e.g., for *L. intracellularis* [20] detection thus affecting the sensitivity of the procedure. However, this drawback could display an advantage for OFs, because inhibitors from the feces are only present in traces here. Moreover, the suitability of OFs to detect and monitor gastrointestinal pathogens would enable a broad overview on circulating pathogens on a farm, in an animal and user-friendly way, as the molecular biological evaluation of OFs can include a whole range of pathogens known to be easily monitored [2, 21]. Examples for testing for nucleic acids of pathogens involving the gastro-intestinal tract (at least partially) from OFs are porcine circovirus 2 (PCV2), PCV3, porcine delta coronavirus (PDCoV), and porcine epidemic diarrhea virus (PEDV) [2, 21–23]. OFs were also used to detect *Yersinia enterocolitica* and others culturally and serologically [10]. However, only limited number of studies are available that have detected antibodies against *L. intracellularis* using OFs [8, 24]. Additionally, only two studies investigated the presence of *B. hyodysenteriae* or *L. intracellularis *in OFs of pigs, respectively [25, 26]. Due to the rationale described above and the very limited number of similar studies available, the need to learn more about the efficiency of *L. intracellularis* and *B. hyodysenteriae* detection from OFs is obvious.

In both farms the animals showed different levels of diarrhea within the study period but the clinical score was not associated with the level of pathogen shedding. This finding might be explained by the pen based fecal score and the pooling of the samples. Especially in terms of OFs, pigs with or without diarrhea might have chewed on the ropes. However, the study design was probably not optimal to state on a correlation between clinical signs and the level of pathogen shedding. Moreover, we did not exclude other pathogens or analyzed food sources that might have been involved in the clinical picture. However, shedding of *L. intracellularis* did not exceed critical levels of 10^7^ *L. intracellularis* per gram of feces, associated with reduced weight gain [27] or10^8^–10^9^/g of Brachyspira, associated with the acute phase of the disease [28]. Concerning the DNA loads in the two sample materials, only a numerical difference was observed. A large-scale examination on this topic as available for PCV2 [3] (OFs vs. serum samples) could allow an adjustment of DNA loads in PFS and OFs by a factor. However, more comparative studies are needed for sufficient accuracy. In this way, it should also be possible to identify critical values for the assessment of the respective pathogens in the existing clinical picture. However, the high agreement resulting from Cohen's kappa values indicates the possibility of simplifying the detectionof *L. intracellularis* and *B. hyodysenteriae* by using OFs. Under respect of the different times of sampling a variability of the interrater reliability for the two sample materials was observed concerning *L. intracellularis* particularly in farm A in week 16 when OFs was more often positive than PFS. The rationale for this observation might be among others the larger number of animals that can be reached by OFS-sampling compared to the pools of individual collected freshly dropped feces. 

## Conclusions

The results of the present study showed the principle proof of concept for using OFs to monitor *B. hyodysenteriae* or *L. intracellularis* in conventional fattening farms under field conditions. Thus, OFs based monitoring of pig herds can be expanded for these pathogens. However, multiple pen testing should be conducted to increase the diagnostic performance and sensitivity.

## Data Availability

The original contributions presented in the study are included in the article, further inquiries can be directed to the corresponding author/s after reasonable request.
